# MYC Promotes LDHA Expression through MicroRNA-122-5p to Potentiate Glycolysis in Hepatocellular Carcinoma

**DOI:** 10.1155/2022/1435173

**Published:** 2022-08-18

**Authors:** Xiaofei Wang, Penghua Zhang, Ke Deng

**Affiliations:** ^1^Department of ICU, Qilu Hospital of Shandong University (Qingdao), Qingdao, 266035 Shandong, China; ^2^Department of Healthy Management Center, Qilu Hospital of Shandong University (Qingdao), Qingdao, 266035 Shandong, China; ^3^Department of General Surgery, Qilu Hospital of Shandong University (Qingdao), Qingdao, 266035 Shandong, China

## Abstract

MYC is a notorious oncogene in a vast network of malignancies, whereas liver-specific microRNA- (miR-) 122-5p is downregulated in hepatocellular cancer (HCC). Here, we studied the possible correlation between these two and their involvement in glycolysis in HCC. MYC was overexpressed in HCC tissues and cells compared to normal liver tissues and normal hepatocytes NHC, which predicted a poor survival of HCC sufferers. Functional assays demonstrated that silencing of MYC inhibited the glycolysis in HCC cells, as evidenced by significantly weaker glucose consumption, lactate production, adenosine triphosphate (ATP) levels, and downregulated HK1 and HK2 protein expression. Moreover, MYC bound to the miR-122-5p promoter and repressed the miR-122-5p expression. Rescue experiments showed that miR-122-5p inhibitor rescued the diminished glycolysis after MYC silencing. In addition, lactate dehydrogenase (LDHA) was identified as a downstream target of miR-122-5p. The overexpression of LDHA mitigated the effects of si-MYC and miR-122-5p mimic on glycolysis of HCC cells, respectively. In conclusion, the MYC/miR-122-5p/LDHA axis modulates glycolysis in HCC cells and possibly affects HCC progression.

## 1. Introduction

The projected 5-year survival for hepatocellular carcinoma (HCC), the major histologic subtype of liver cancer, is only 18%, which fundamentally reflects that only 30%–40% of patients are diagnosed at a relative early stage and are suitable for surgical resection, ablation, or liver transplantation [1]. Therefore, therapeutic and prognostic targets are necessary to be explored currently to improve the survival of HCC patients in the decades to come. Normal cells break down glucose or glycogen into lactate while producing a small amount of adenosine triphosphate (ATP) under anaerobic or hypoxic conditions [2]. However, due to the energy demand for rapid cell multiplication, tumor cells decompose glucose into lactate through glycolysis, known as the Warburg effect, which is why glycolysis is particularly important in tumor cell metabolism [3].

Since the 1980s, the focus of MYC, the gene encodes c-MYC, b-MYC, l-MYC, n-MYC, and s-MYC, has been concentrated on the carcinogenesis of HCC, including the cell proliferation, growth, cell cycle, and differentiation [4]. Interestingly, MYC has been revealed to suppress expression profile of primary and mature microRNA- (miR-) 122 in hepatic cells [5]. Moreover, MYC-driven liver tumors have been documented to be related to boosted glucose catabolism through glycolysis and augmented pyruvate kinase activity [6]. Importantly, as the richest liver-specific miRNA, miR-122 is participated in several physiological processes in hepatic function and liver pathology, and miR-122 downregulation in HCC has been validated [7]. A panel consisting of miR-122-5p + miR-486-5p + miR-142-3p distinguished HCC from cirrhosis (AUC = 0.94; sensitivity = 80%, specificity = 95%; *p* < 0.001) [8]. In addition, the ectopic expression of miR-122-5p constrained proliferation, invasion, and metastasis, whereas stimulated apoptosis of HCC cells [9]. Restoring the expression of miR-122-5p inhibited the hallmark glycolytic enzymes and ultimately the metabolic activity of HCC cells [10]. Therefore, we wondered if miR-122-5p, reduced by MYC transcriptionally, could also be engaged in the mediation of glycolysis in HCC. The lactate dehydrogenase (LDHA), an important enzyme of the glycolysis, catalyzes the generation of lactate, and its high expression has been verified in a myriad of cancers, which is related to malignant progression [11]. Moreover, LDHA has been found to be targeted by miR-100-5p and miR-142-3p, thus involving in glycolysis and proliferation in HCC [12, 13]. In this study, the possible interaction between miR-122-5p and LDHA was probed, and the potential role of the MYC/miR-122-5p/LDHA axis for modulating the glycolysis in HCC cells was demonstrated.

## 2. Materials and Methods

### 2.1. Clinical Tissue Specimens

Fifty-nine patients with HCC admitted to Qilu Hospital of Shandong University from June 2007 to June 2008 were included in this study with the approval by the Research Ethics Committee of Qilu Hospital of Shandong University, and written informed consent was acquired from all participants. Inclusion criteria were as follows: diagnosis of HCC confirmed by imaging and pathological biopsy, patients with expected survival time > 3 months, complete clinical data and good compliance, and did not receive targeted antitumor therapies, including surgery, radiotherapy, and chemotherapy prior to enrollment. Patients who were not accompanied by their family members at the time of admission, with autoimmune system deficiencies or infections, and those refused to provide specimens were excluded. HCC and paracancerous tissues (more than 5 cm away from the cancerous tissue) were collected.

### 2.2. Cell Lines and Cell Culture

Human normal hepatocyte NHC (Jining Shiye, Shanghai, China) and HCC cell lines MHCC97H (Shanghai Zhong Qiao Xin Zhou Biotechnology Co., Ltd., Shanghai, China), Huh7, and Hep3B (National Collection of Authenticated Cell Cultures, Shanghai, China) were included in this study. The Hep3B cells were cultured in minimal essential medium (Gibco, Carlsbad, CA, USA) plus 10% FBS (Gibco), 50 U/mL penicillin (Gibco), and 100 *μ*g/mL streptomycin (Gibco). NHC, MHCC97H, and Huh7 cells were cultured with Dulbecco's Modified Eagle's Medium (DMEM). The culture condition was at 37°C in a 5% CO_2_ cell incubator (Thermo Fisher Scientific Inc., Waltham, MA, USA). The media were renewed daily. After 3-4 d, the cells were subcultured, and the cells in logarithmic growth phase and in good growth condition were selected for the following assays.

### 2.3. Cell Transfection

Logarithmically growing MHCC97H and Huh7 cells were seeded in 6-well culture plates at 1 × 10^5^ cells/well. RPMI-1640 medium free of serum and penicillin/streptomycin was used the day before transfection. Transfections were performed strictly according to the Lipofectamine 2000 instructions. Small interfering RNA (si) targeting MYC (si-MYC-1 and si-MYC-2, 2 ng), miR-122-5p inhibitor, miR-122-5p mimic, oe-LDHA, or negative control (NC, Shanghai GenePharma Co., Ltd., Shanghai, China) were centrifuged, mixed with 170 *μ*L PBS (Thermo Fisher), and left for 5 min. After the addition of 8 *μ*L Lipofectamine 2000 (11668019, Thermo Fisher), the mixture was left at room temperature for 20 min. The above mixture was loaded to a six-well plate and gently mixed. After 8 h of transfection, the cells were grown in medium plus 10% FBS (Gibco) for 48 h, followed by further assays.

### 2.4. RT-qPCR

After a 48-h transfection, total RNA was isolated from each group of cells using TRIzol (Thermo Fisher). cDNA was synthesized by reverse transcription using TaqMan MicroRNA Assay Kit (Thermo Fisher). Fluorescent qPCR was performed using TB® Premix Ex TaqTM II kit (RR820A, Takara, Dalian, Liaoning, China) on an ABI PRISM® 7300 (ABI Company, Oyster Bay, N.Y., USA) system. The expression was normalized to U6 or *β*-actin to produce a 2^−*ΔΔ*Ct^ value for the relative expression. The primers are displayed in Table 1.

### 2.5. Western Blot Assays

Total protein was harvested from the cells using RIPA lysis buffer (R0010, Solarbio, Beijing, China) containing phenylmethanesulfonyl fluoride after 48 h of cell transfection. A BCA kit (Thermo Fisher) was utilized to assess the protein concentration. The samples were mixed with the loading sample buffer and bathed for 10 min at 100°C. Then, 50 *μ*g protein sample was added, and electrophoresis was performed at a constant voltage of 70 V for 3 h. The proteins were transferred to PVDF membranes (Millipore Corp, Billerica, MA, USA) which were blocked with 5% skimmed milk powder at 4°C for 120 min and probed with the primary rabbit antibodies to MYC (ab32072, 1 : 1,000, Abcam, Cambridge, UK), LDHA (AF0216, 1 : 1,000, Beyotime, Shanghai, China), HK1 (AF1726, 1 : 1,000, Beyotime), HK2 (ab209847, 1 : 5,000, Abcam), and *β*-actin (ab115777, 1 : 200, Abcam) at 4°C for one night and with HRP-labeled goat anti-rabbit IgG antibody (SE134, 1 : 5,000, Solarbio) for 2 h. Electrochemiluminescence (ECL) kit (BB-3501, Bestbio, Shanghai, China) was applied to measure ECL fluorescence. Then, the membrane was dropped with a mixture of solutions A and B (200 *μ*L) in the dark. Then, the Bio-Rad image analysis system (Bio-Rad Laboratories, Hercules, CA, USA) was applied. The relative protein expression was analyzed using ImageJ (NIH, Bethesda, MD, USA) software, which was expressed as the grayscale value of the corresponding protein band/grayscale value of the *β*-actin protein band.

### 2.6. ELISA

ELISA kits for LDHA and LDHB were purchased from GenScript (Nanjing, Jiangsu, China) and Abcam, respectively. The antigen was diluted with the coating solution as described in the kit and incubated overnight at 4°C in the 96-well plate. The 96-well plate was sealed after discarding the coating solution and adding the sealing solution. The sample or standard was added for a 2-h incubation, followed by the incubation with the secondary antibody for 40 min. After washing, TMB was added for color development, and finally, the reaction was terminated by adding termination solution. The optical density (OD) at 450 nm was measured in a microplate reader.

### 2.7. Chromatin Immunoprecipitation (ChIP)

The cells were spread in six-well plates and fixed with 4% formaldehyde. The proteins and DNA were crosslinked. The cells were lysed with cell lysis solution, and chromatin was sonicated and incubated overnight with MYC (ab32072, 1 : 500, Abcam) and IgG (ab172730, 1 : 500, Abcam) antibodies, respectively, for one night. The magnetic beads were supplemented to capture the protein DNA-bound complexes, and then 5 mmol/L NaCl was supplemented to de-crosslink the DNA. The DNA was recovered, and the enrichment of miR-122-5p promoter region bound in the complex was detected by fluorescence qPCR.

### 2.8. Dual-Luciferase Assays

The wild and mutant reporter plasmids for LDHA (wt-LDHA, mut-LDHA) were designed by GenePharma (Shanghai, China). NC inhibitor or miR-122-5p inhibitor was co-transfected with wt-LDHA or mut-LDHA into 293 T cells, respectively. The 293 T cells were cultured for 2 d. Alterations in luciferase activity were detected as per the procedure in the Dual Luciferase Assay Kit (E1910, Promega Corporation, Madison, WI, USA). Fluorescence intensity was assessed with a GLomax 20/20 luminometer fluorescence detector (E5311, Promega).

### 2.9. Glucose Consumption and Lactate Production

Colorimetric assay was used for the assessment of glucose and lactate concentration in cell culture medium. After a 48 h transfection, 1 mL supernatant was gently aspirated into a new Eppendorf (EP) tube. Fresh complete medium (1 mL) was aspirated into a centrifuge tube as a blank group. The sample was centrifuged at 2500 rpm for 10 min, and the supernatant was taken for determination. The reagents and samples were added step by step following the instructions of the kit (KA4086, Abnova, Walnut, CA, USA), mixed, and water-bathed at 37°C for 15 min, and the OD values of each well were assessed. The lactate production was measured in the same way (BC2230, Solarbio) except for no centrifugation.

### 2.10. Assessment of Cellular ATP Content

The cellular ATP levels were assessed using chemiluminescence assay. After a 48 h transfection, the cells in the 6-well plate were lysed with ATP lysis solution and centrifuged at 4°C for 15 min at 12,000 rpm. The supernatant was the ATP assay sample, and the ATP assay solution in the ATP assay kit (S0026, Beyotime) was diluted at 1 : 9 with the ATP dilution solution, which is the ATP assay working solution. The ATP assay working solution was supplemented to the EP tube before the assay to consume the background ATP in the tube, and the mixture of ATP sample and ATP assay solution was supplemented to the EP tube at 1 : 1. The luminescence value was detected on the chemiluminescence instrument after 5 s of reaction.

### 2.11. CCK-8 Assay

After a 48 h transfection, cells in the logarithmic growth phase were prepared into cell suspension at 1 × 10^4^ cells/mL using DMEM plus 10% FBS and seeded into 96-well culture plates. According to the experimental grouping, 8 wells were set up for each group, and 100 *μ*L cell suspension was supplemented into each well for an incubation in a 5% CO_2_ cell culture chamber at 37°C. At the 24^th^ h, 10 *μ*L CCK8 (Sigma-Aldrich, St Louis, MO, USA) was supplemented to each well, and the incubation continued for 1 h. The OD values were read at 450 nm on a microplate reader.

### 2.12. Trypan Blue Assay

After the cells were transfected for 48 h, the cells were detached with trypsin. A total of 1 × 10^3^ cells were seeded into a 96-well plate, collected after detachment for 24 h, and resuspended with culture medium. The cells were stained with trypan blue (C0011, Beyotime), and the stained cells were added to the cell counting plate for counting under the microscope.

### 2.13. Transwell Assay

The migration and invasion of the cells were assayed with the Transwell culture system. A permeable membrane coated with Matrigel (BD Biosciences, San Jose, CA, USA) was used. The chamber was rehydrated for 120 min in a 37°C incubator with serum-free medium. The apical chamber was supplemented with the cell suspension (1 × 10^5^ cells in 200 *μ*L). The medium containing 10% FBS (500 *μ*L) was added as a chemical attractant into the basolateral chamber. After incubation at 37°C for 1 d, the cells on the lower surface were fixed with formaldehyde for 5 min at room temperature, treated with crystal violet for 20 min, and counted under a microscope. Migration assays were carried out in the same way as invasion assays, without Matrigel coating before inoculation of cells in the apical chamber.

### 2.14. Statistical Analyses

Statistical analyses were implemented with SPSS software (21.0; SPSS, Chicago, IL, USA). Results are presented as the mean ± SD from three individual experiments for each group. The differences between two groups were estimated by the paired or unpaired *t*-test; the differences among more than two groups were estimated by one-way or two-way ANOVA, followed by Tukey's post hoc test. Statistical significance was defined as *p* < 0.05.

## 3. Results

### 3.1. MYC Is Highly Expressed in HCC

**Figure 1:**
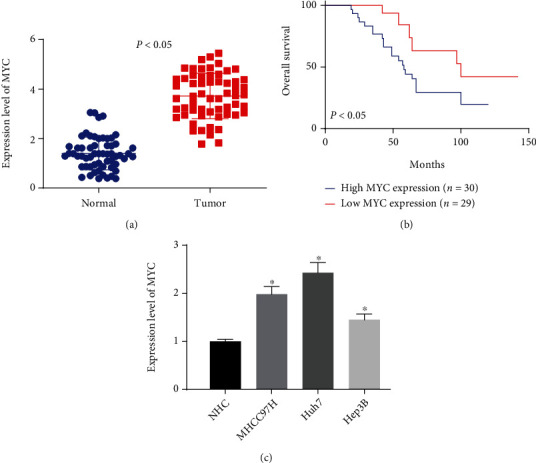
MYC is overexpressed in HCC. (a) MYC expression in HCC tissues using RT-qPCR. (b) The correlation between MYC expression and survival of HCC patients. (c) Detection of the MYC expression in HCC cells by RT-qPCR. ^∗^*p* < 0.05 vs. normal tissues or NHC cells. Statistical results were presented by mean ± SD. Paired *t*-test and one-way ANOVA were used for comparisons. Each reaction was run in triplicate.

To investigate the changes of the MYC expression in HCC, 59 pairs of HCC and matched normal tissues were collected, and the correlations between MYC expression and clinicopathology are displayed in Table 2. The MYC expression was not significantly correlated with age and lymph node metastasis of HCC patients but was significantly linked to tumor diameter, differentiation level, and TNM stage. RT-qPCR was utilized to test the MYC expression, which presented that MYC was much higher in HCC tissues than that in normal tissues (Figure 1(a)). By analyzing the correlation between the MYC expression in HCC tissues and the prognostic survival of HCC patients, we observed that the patients with high MYC expression had much shorter survival, while those with low MYC expression had significantly longer survival (Figure 1(b)). Compared with human normal hepatocytes NHC cells, the MYC expression was significantly higher in HCC cells MHCC97H, Huh7, and Hep3B (Figure 1(c)). Huh7 and MHCC97H cells with the relative high expression were chosen for following experiments.

### 3.2. Silencing of MYC Constrains Glycolysis in HCC Cells

**Figure 2:**
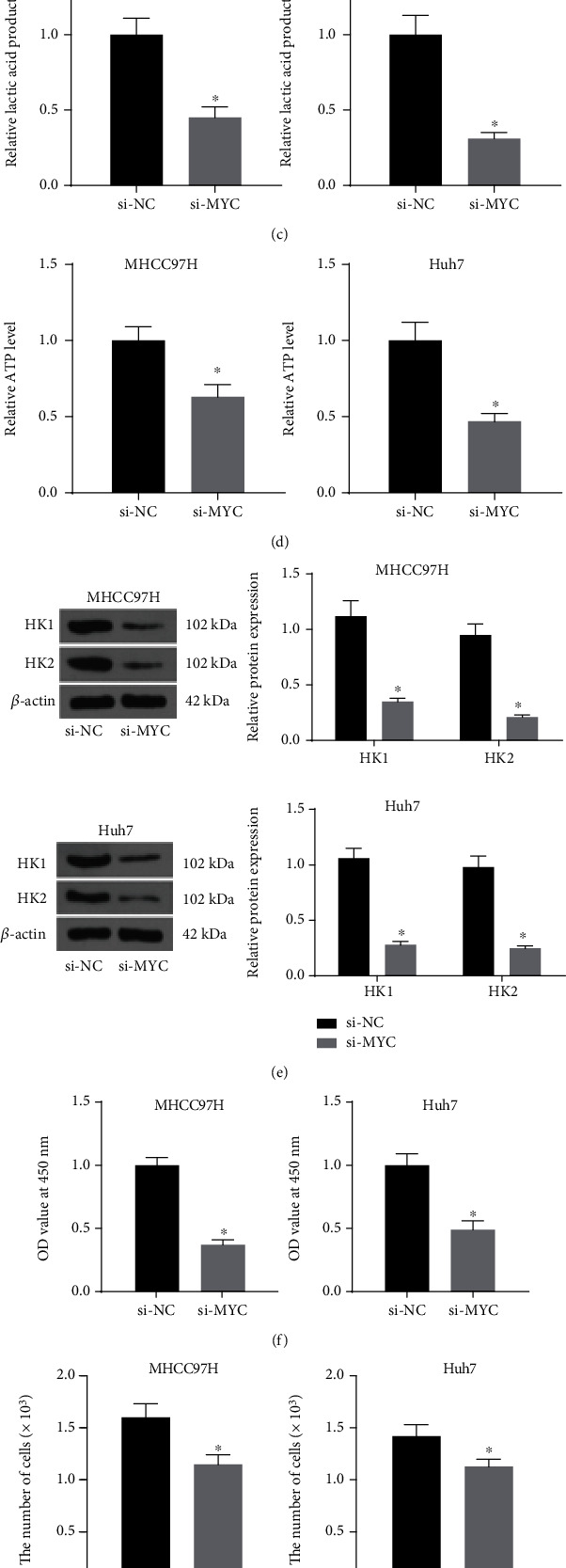
Silencing of MYC inhibits glycolysis in HCC cells. (a) The transfection efficiency of si-MYC in MHCC97H and Huh7 cells measured using western blot. The changes in glucose consumption (b), lactate production (c), and ATP levels (d) in MHCC97H and Huh7 cells measured by their respective kits. (e) The protein expression of HK1 and HK2 in MHCC97H and Huh7 cells assessed using Western blot. (f) The proliferation of MHCC97H and Huh7 cells measured using CCK8. (g) Detection of cell proliferation by trypan blue assay. (h) The aggressiveness of MHCC97H and Huh7 cells examined using Transwell assays. ^∗^*p* < 0.05 vs. si-NC. Statistical results were presented by mean ± SD. Unpaired *t*-test and one-way or two-way ANOVA were used for comparisons. Each reaction was run in triplicate.

The previous results exhibited that MYC was overexpressed in HCC cells; so, MYC was knocked down in MHCC97H and Huh7 cells. The interfering efficiency of si-MYC #1 and #2 was assessed by Western blot.  Figure 2(a) shows that the MYC protein expression was appreciably downregulated after si-MYC compared with si-NC, and we chose the one sequence with better effect for the experiment. As expected, the glucose consumption and lactate production were drastically reduced by si-MYC (Figures 2(b) and 2(c)). Consistently, cellular ATP levels were significantly lower in si-MYC-treated cell (Figure 2(d)). Western blot was applied to assess the protein expression of HKs. The protein expression of HK1 and HK2 was significantly downregulated after MYC knockdown (Figure 2(e)). The cell proliferation was evaluated by CCK8 and trypan blue method. As shown in Figures 2(f) and 2(g), the cell growth was significantly delayed in the si-MYC group. Moreover, the invasion and migration of cells in the si-MYC group were significantly hampered as well (Figure 2(h)). In summary, silencing of MYC inhibited the glycolysis in HCC cells and suppressed cell growth.

### 3.3. MYC Expedites Glycolysis in HCC Cells by Binding to miR-122-5p

**Figure 3:**
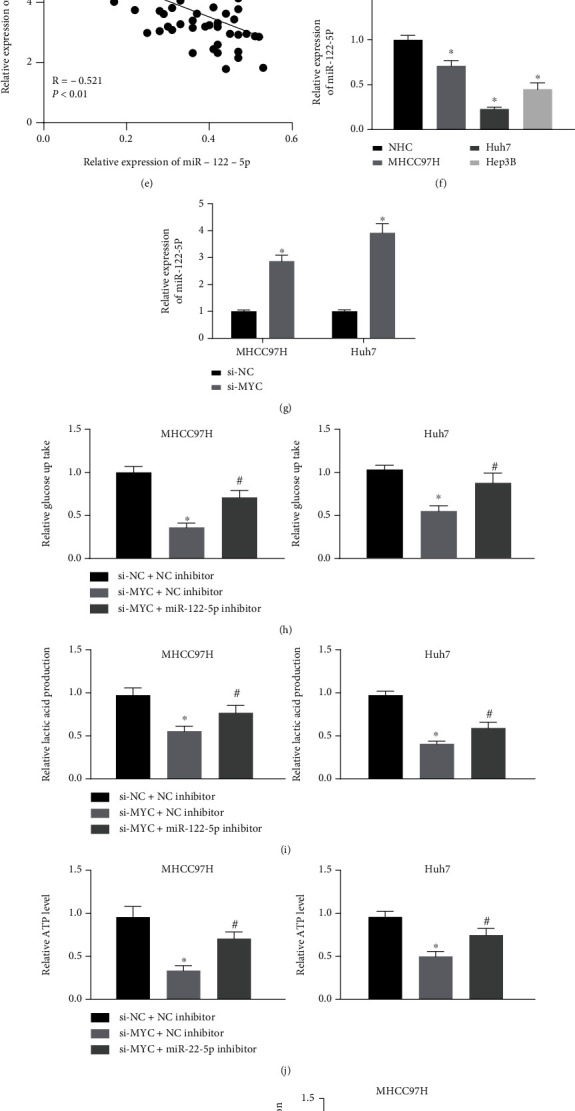
MYC enhances glycolysis in HCC cells by interacting with miR-122-5p. (a) Screening of miR-122-5p upstream factors on the bioinformatics website. (b) Detection of MYC binding to miR-122-5p promoter examined using ChIP. (c) Detection of the miR-122-5p expression in clinical tissues using RT-qPCR. (d) The correlation between miR-122-5p expression and survival of patients with HCC. (e) The correlation between miR-122-5p and MYC expression in HCC tissues. (f) The miR-122-5p expression in NHC and HCC cells by RT-qPCR. (g) Detection of the miR-122-5p expression by RT-qPCR after depletion of MYC in MHCC97H and Huh7 cells. (h)–(j) The changes in glucose consumption (h), lactate production (i), and ATP contents (j) in MHCC97H and Huh7 cells assessed by their respective kits. (k) The protein expression of HK1 and HK2 in MHCC97H and Huh7 cells evaluated using Western blot. ^∗^*p* < 0.05 vs. IgG, NHC cells, si-NC, or si-NC + NC inhibitor; #*p* < 0.05 vs. si-MYC + NC inhibitor. Statistical results were presented by mean ± SD. Paired *t*-test and one-way or two-way ANOVA were used for comparisons. Each reaction was run in triplicate.

In conjunction with previous literature, it was reported that MYC binds to the miR-122 promoter to transcriptionally repress the miR-122 expression [5]. TransmiR v2.0 (http://www.cuilab.cn/transmir) was applied to screen for transcriptional regulators of miR-122. A total of 23 candidates were screened (Figure 3(a)). The binding of MYC to the miR-122-5p promoter was assessed by ChIP. Compared with IgG, significantly more MYC was enriched on the miR-122-5p promoter (Figure 3(b)). The expression of miR-122-5p in HCC tissues was detected by RT-qPCR. As shown in Figure 3(c), miR-122-5p was lowly expressed in HCC tissues. By analyzing the correlation between the expression of miR-122-5p in HCC tissues and the survival of HCC patients, we observed that the survival of patients with the high miR-122-5p expression was longer, while the survival of patients with the low miR-122-5p expression was significantly shorter (Figure 3(d)). In Figure 3(e), the MYC expression was observed to show a converse correlation with the miR-122-5p expression in HCC. The expression of miR-122-5p in NHC cells and HCC cells was measured using RT-qPCR. miR-122-5p was poorly expressed in HCC cells (Figure 3(f)). The miR-122-5p expression in response to si-MYC in MHCC97H and Huh7 cells was evaluated using RT-qPCR. In Figure 3(g), miR-122-5p levels were significantly restored following si-MYC.

Subsequently, the HCC cells were co-transfected with si-NC + NC inhibitor, si-MYC + NC inhibitor, or si-MYC + miR-122-5p inhibitor. Relative to the si-NC + NC inhibitor group, the glucose consumption was considerably diminished in the si-MYC + NC inhibitor group, while the repressive effects of si-MYC on glucose consumption was mitigated by miR-122-5p inhibitor (Figure 3(h)). Consistent trends were observed in the lactate production and cellular ATP levels (Figures 3(i) and 3(j)). Western blot was finally performed to measure the protein expression of HKs. The protein expression of HK1 and HK2 was significantly downregulated by si-MYC, which was restored by miR-122-5p inhibitor (Figure 3(k)).

### 3.4. miR-122-5p Represses Glycolysis in HCC Cells through Targeting LDHA

**Figure 4:**
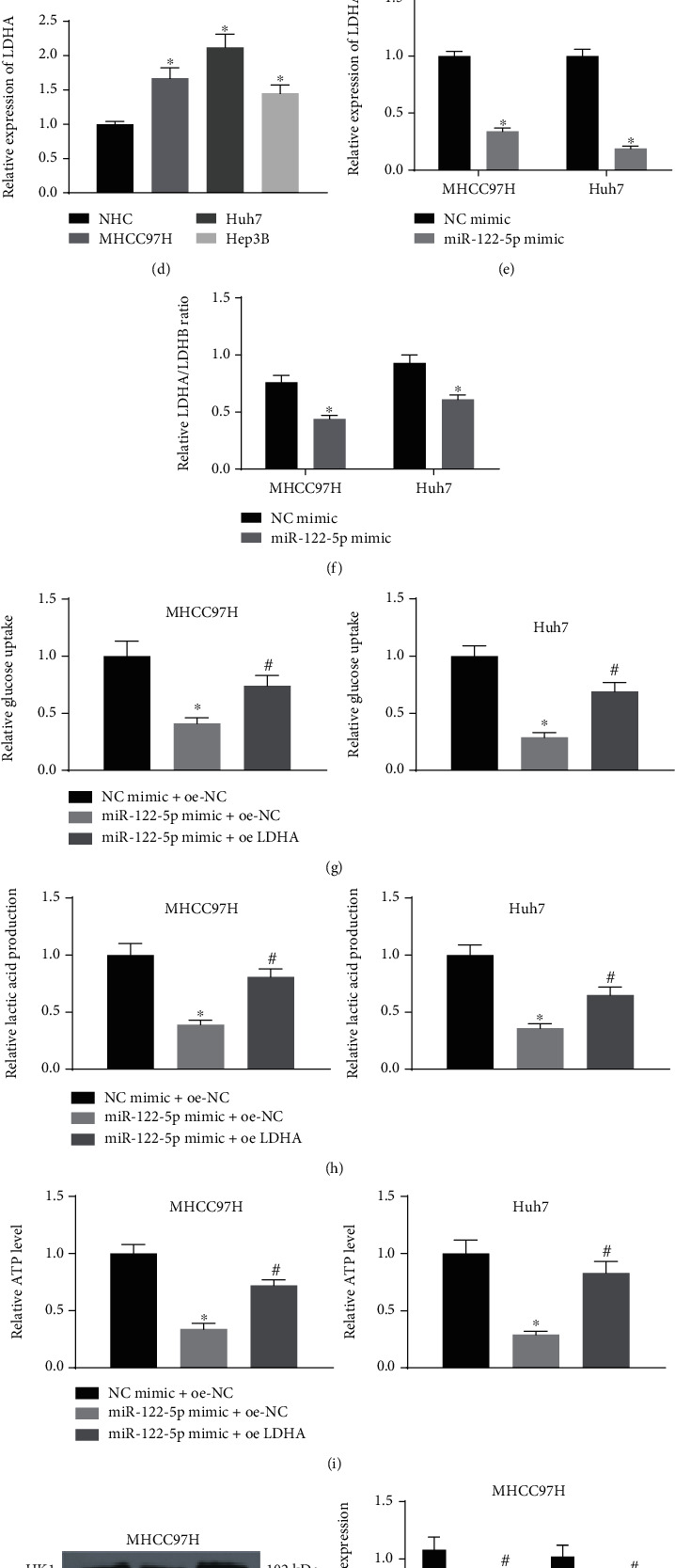
miR-122-5p hinders glycolysis in HCC cells through targeting LDHA. (a) Prediction of the targeting sites between miR-122-5p and LDHA 3′UTR. (b) The binding relation between miR-122-5p and LDHA verified using dual-luciferase reporter assays. (c) LDHA expression in clinical tissues using RT-qPCR. (d) LDHA expression in NHC cells and HCC cells MHCC97H, Huh7, Hep3B using RT-qPCR. (e) LDHA expression by RT-qPCR and western blot after restoration of miR-122-5p in MHCC97H and Huh7 cells. (f) Detection of LDHA/LDHB ratio in MHCC97H and Huh7 cells using ELISA. (g)–(i) Glucose consumption (g), lactate production (h), and ATP contents (i) in MHCC97H and Huh7 cells measured by their respective kits. (j) The protein expression of HK1 and HK2 in MHCC97H and Huh7 cells evaluated using Western blot. ^∗^*p* < 0.05 vs. NC mimic, NHC cells, or NC mimic + oe-NC; #*p* < 0.05 vs. miR-122-5p mimic + oe-NC. Statistical results were presented by mean ± SD. Paired *t*-test and one-way or two-way ANOVA were used for comparisons. Each reaction was run in triplicate.

When investigating the downstream regulatory mechanism of miR-122-5p, we found that miR-122-5p targeted LDHA mRNA 3′UTR (Figure 4(a)). Dual-luciferase analysis verified that the miR-122-5p overexpression significantly repressed LDHA-wt luciferase activity (Figure 4(b)). The expression of LDHA in clinical tissues, NHC and HCC cell lines MHCC97H, Huh7, and Hep3B was detected by RT-qPCR. LDHA was considerably upregulated in both HCC tissues and cell lines (Figures 4(c) and 4(d)). The LDHA expression profile was assessed by RT-qPCR and western blot after the overexpression of miR-122-5p in Huh7 and MHCC97H cells. As displayed in Figure 4(e), LDHA mRNA and protein expression was appreciably downregulated following miR-122-5p mimic. Moreover, the ratio of LDHA/LDHB was reduced following miR-122-5p mimic, as evidenced by ELISA (Figure 4(f)).

Also, we transfected Huh7 and MHCC97H cells with NC mimic + oe-NC, miR-122-5p mimic + oe-NC, or miR-122-5p mimic + oe-LDHA. We found that miR-122-5p mimic remarkably weakened the glucose consumption, lactate production, and ATP contents in HCC cells, which were all restored by the LDHA overexpression (Figures 4(g)–4(i)). Additionally, the examination of HKs using western blot revealed that miR-122-5p mimic reduced the HK1 and HK2 expression, while the LDHA overexpression partially reversed the effects of miR-122-5p mimic on the HK1 and HK2 expression (Figure 4(j)). In summary, miR-122-5p reduces the glycolysis in HCC cells through targeting LDHA.

### 3.5. MYC/miR-122-5p/LDHA Axis Regulates the Glycolysis in HCC Cells

**Figure 5:**
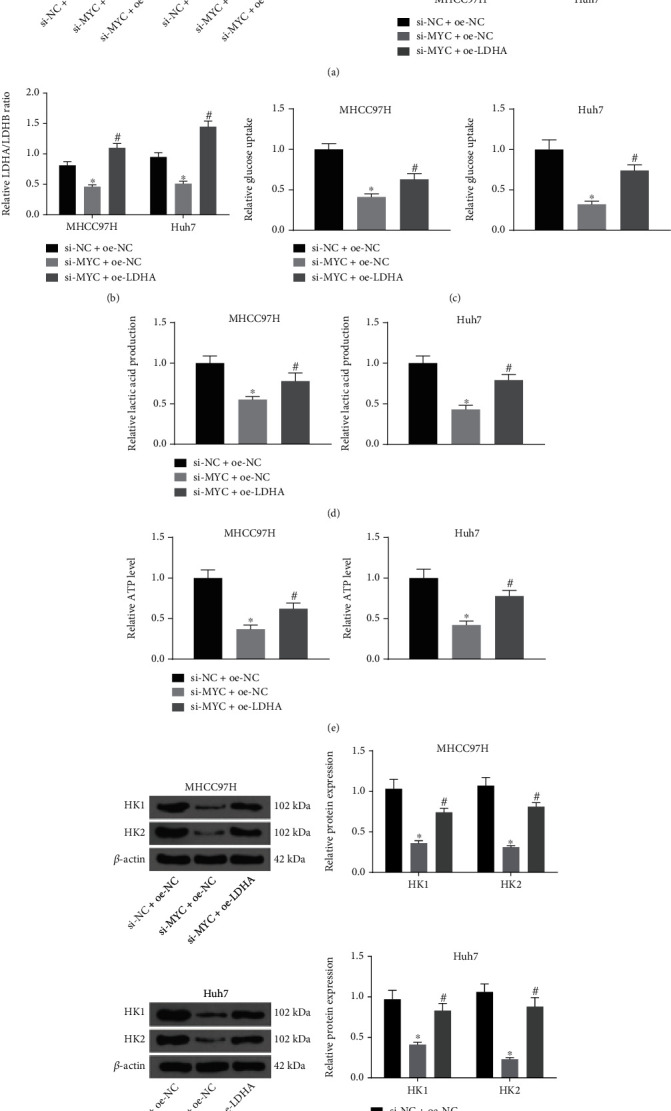
MYC/miR-122-5p/LDHA axis regulates the glycolysis in HCC cells. (a) Detection of MYC and LDHA expression in MHCC97H and Huh7 cells by Western blot. (b) Detection of LDHA/LDHB ratio in MHCC97H and Huh7 cells using ELISA. (c)–(e) The changes in glucose consumption (c), lactate production (d), and ATP contents (e) in MHCC97H and Huh7 cells measured by their respective kits. (f) The protein expression of HK1 and HK2 in MHCC97H and Huh7 cells measured using Western blot. ^∗^*p* < 0.05 vs. si-NC + oe-NC; #*p* < 0.05 vs. si-MYC + oe-NC. Statistical results were presented by mean ± SD. One-way or two-way ANOVA was used for comparisons. Each reaction was run in triplicate.

To verify whether there is a MYC/miR-122-5p/LDHA axis in HCC cell, we co-transfected HCC cells with si-NC + oe-NC, si-MYC + oe-NC, or si-MYC + oe-LDHA. Western blot was implemented to assess the MYC and LDHA expression following co-transfection. Compared with si-NC + oe-NC, the MYC expression was downregulated in si-MYC + oe-NC and si-MYC + oe-LDHA groups, and the LDHA expression was reduced in si-MYC + oe-NC. Relative to the si-MYC + oe-NC group, the LDHA expression was upregulated in the si-MYC + oe-LDHA group (Figure 5(a)). Regarding the ratio of LDHA/LDHB, silencing of MYC reduced the LDHA/LDHB ratio, which was restored by oe-LDHA (Figure 5(b)). Still, the alleviating effects of si-MYC on glucose consumption, lactate production, and ATP contents were rescued by oe-LDHA (Figures 5(c)–5(e)). As shown in Figure 5(f), the downward trends of the HK1 and HK2 expression in HCC cells with si-MYC were hampered by oe-LDHA.

## 4. Discussion

Aerobic glycolysis, firstly observed in HCC, serves as a hallmark of liver cancer and holds accountable for the modulation of immune evasion, metastasis, and angiogenesis, as well as drug resistance in HCC [14]. To provide insights into the molecular mechanism underlying glycolysis, in the current report, we elucidated the function of MYC/miR-122-5p/LDHA in HCC. The expression of MYC was promoted in HCC tissues and cells, and that depletion of MYC remarkably repressed the glycolysis, proliferation, migration, and invasion of HCC cells by interacting with miR-122-5p. Moreover, we found that LDHA which was increased in HCC and a putative target of miR-122-5p rescued the inhibiting effects of si-MYC and miR-122-5p mimic on the glycolysis of HCC cells. Taken together, our study demonstrates a crucial role of MYC/miR-122-5p/LDHA in glycolysis, offering avenues for future therapy development in HCC.

MYC, a well-characterized driving force in a vast network of organs including the liver, is a transcription factor that stimulates cellular transformation and tumor progression, and the significant role of MYC in hepatocarcinogenesis has been established unquestionably by the finding that MYC upregulation is efficient enough to initiate HCC development in mice [15]. In the present report, we not only substantiated that overexpression of MYC was correlated with dismal prognosis of HCC patients but also demonstrated that silencing of MYC hampered the glycolysis, migration, invasion, and growth of HCC cells. Similarly, downregulation of MYC decreased ATP production in T cell receptor-stimulated T cells [16]. The potential of MYC for regulating glycolysis in cancers has been indicated in B-cell acute lymphocytic leukemia, colorectal cancer, and breast cancer [17–19]. Furthermore, MYC is currently considered as “undruggable,” making it a strong driver to identify downstream effectors of MYC, whose suppression is detrimental for the growth of MYC-driven tumors [20].

Nakao et al. reported that MYC can curb the miR-122 expression by physically interacting with its promoter and by reducing the expression of HNF3b [21]. Mechanistically, PRMT5 has been revealed as an epigenetic executer of MYC, contributing to suppression of the transcription of downstream genes that support carcinogenesis in HCC, highlighting a direction for MYC-driven HCC via PRMT5 inhibition [22]. In the present study, using bioinformatics tools, we found that MYC is one of the 23 upstream factors of miR-122-5p. The downregulation of miR-122-5p in HCC exhibited diagnostic values, thereby miR-122-5p may act as a tumor inhibitor [23]. Our prognostic analysis also revealed that patients with higher miR-122-5p benefited from prolonger survival time. Additionally, miR-122-5p inhibitor mitigated the inhibitory effects of si-MYC on glycolysis. In line with our observation, the ectopic expression of miR-122-5p blocked the promoting effect of long noncoding RNA (lncRNA) SOX2OT on glucose uptake, glycolysis, and lactate production in HCC cells [24].

Later, we found that LDHA is a downstream effector of miR-122-5p. Subsequently, the upregulation of LDHA has been corroborated in HCC, which was in agreement with other two studies where the LDHA overexpression was closely correlated with differentiation status, vascular invasion, TNM stage, and the overall survival of HCC [25, 26]. LDHA has been also reported to be targeted by different miRNAs in various cancers, including osteosarcoma [27] and bladder cancer [28]. Also, knockdown of lncRNA HOXB-AS3 hampered glycolysis of epithelial ovarian cancer by decreasing the LDHA expression [29]. In addition, lncRNA SNHG7, a transcriptional target of MYC, positively regulated LDHA level in glycolysis in breast cancer as well [30]. Interestingly, Sp1 binding sites, as positive regulatory elements in the LDHA promoter, were participated in promoting LDHA transcription in testicular tumor cells [31]. In this investigation, we exhibited that miR-122-5p directly targeted LDHA, and that MYC could also modify the LDHA expression by interacting with miR-122-5p. Additionally, both miR-122-5p and MYC regulated glycolysis of HCC cells through LDHA. It might be possible that there are some other upstream regulators of miR-122-5p in HCC, and upcoming investigations are necessary to test this. Furthermore, MYC regulates the gene expression either directly, such as glycolytic genes including LDHA, or indirectly, such as repression of miRNAs [32]. Additional studies are warranted to verify that MYC promotes the LDHA expression through miR-122-5p.

## 5. Conclusion

In summary, with a combination of *in vitro* HCC cells as well as human HCC samples, we provided strong evidence that MYC/miR-122-5p/LDHA plays a major role in the glycolysis of HCC. Suppression of MYC or overexpression of miR-122-5p could hamper HCC cell glycolysis, thus serving as molecular targets to develop effective therapy.

## Figures and Tables

**Table 1:** The primer sequence of RT-qPCR.

Genes	Primer sequence
MYC	F: 5′-AGAGTTTCATCTGCGACCCG-3′
	R: 5′-AAGCCGCTCCACATACAGTC-3′
miR-122-5p	F: 5′-GTGACAATGGTGGAATGTGG-3′
	R: 5′-AAAGCAAACGATGCCAAGAC-3′
LDHA	F: 5′-AGCCCGATTCCGTTACCTAATG-3′
	R: 5′-ACCTGCTTGTGAACCTCTTTCC-3′
*β*-Actin	F: 5′-CACCATTGGCAATGAGCGGTTC-3′
	R: 5′-AGGTCTTTGCGGATGTCCACGT-3′
U6	F: 5′-GCTTCGGCAGCACATATACTAAAAT-3′
	R: 5′-CGCTTCACGAATTTGCGTGTCAT-3′

Note: RT-qPCR: reverse transcription quantitative PCR; miR-122-5p: microRNA-122-5p; LDHA: lactate dehydrogenase A; F: forward; R: reverse.

**Table 2:** Relationship between MYC levels and clinicopathological features of HCC patients.

Clinicopathological features	*n*	MYC expression	
		Mean ± standard deviation	*p*
Sex			>0.05
Male	40	3.63 ± 1.02	
Female	19	3.91 ± 0.59	
Age (years)			>0.05
≤45	35	3.56 ± 0.95	
>45	24	3.93 ± 0.83	
Tumor diameter (cm)			< 0.05
≤ 4	27	3.24 ± 0.85	
> 4	32	4.29 ± 0.61	
Tissue differentiation			< 0.05
Poor	11	4.65 ± 0.58	
Moderate	23	3.97 ± 0.69	
Well	25	3.08 ± 0.74	
Lymph node metastasis			>0.05
Yes	13	3.51 ± 0.65	
No	46	3.83 ± 0.97	
TNM staging			< 0.05
I	19	3.09 ± 0.85	
II	25	3.72 ± 0.73	
IIIa	15	4.53 ± 0.65	

Note: HCC: hepatocellular cancer; TNM: tumor, node, metastases.

## Data Availability

The data used to support the findings of this study are included within the article.
